# Decision-Making Process Reported by Medicare Patients Who Had Coronary Artery Stenting or Surgery for Prostate Cancer

**DOI:** 10.1007/s11606-012-2009-5

**Published:** 2012-02-28

**Authors:** Floyd J. Fowler, Patricia M. Gallagher, Julie P. W. Bynum, Michael J. Barry, F. Leslie Lucas, Jonathan S. Skinner

**Affiliations:** 1 Center for Survey Research, University of Massachusetts Boston, 100 Morrissey Blvd., Boston, MA 02125 USA; 2 Foundation for Informed Medical Decision Making, Boston, MA USA; 3 Department of General Internal Medicine, Dartmouth Hitchcock Medical Center, Hanover, NH USA; 4 General Medicine Division, Massachusetts General Hospital, Boston, MA USA; 5 Center for Outcomes Research, Maine Medical Center, Portland, ME USA; 6 Department of Economics, Dartmouth College, Hanover, NH USA; 7 The Dartmouth Institute, Dartmouth College, Hanover, NH USA

**Keywords:** shared decision making, decision quality, patient-centered care

## Abstract

**Background:**

Patients facing decisions should be told about their options, have the opportunity to discuss the pros and cons, and have their preferences reflected in the final decision.

**Objectives:**

To learn how decisions were made for two major preference-sensitive interventions.

**Design:**

Mail survey of probability samples of patients who underwent the procedures.

**Participants:**

Fee-for-service Medicare beneficiaries who had surgery for prostate cancer or elective coronary artery stenting in the last half of 2008.

**Main measurements:**

Patients’ reports of which options were presented for serious consideration, the amount of discussion of the pros and cons of the chosen option, and if they were asked about their preferences.

**Results:**

The majority (64%) of prostate cancer surgery patients reported that at least one alternative to surgery was presented as a serious option. Almost all (94%) said they and their doctors discussed the pros, and 63% said they discussed the cons of surgery “a lot” or “some”. Most (76%) said they were asked about their treatment preferences. Few who received stents said they were presented with options to seriously consider (10%). While most (77%) reported talking with doctors about the reasons for stents “a lot” or “some”, few (19%) reported talking about the cons. Only 16% said they were asked about their treatment preferences.

**Conclusions:**

While prostate cancer surgery patients reported more involvement in decision making than elective stent patients, the reports of both groups document the need for increased efforts to inform and involve patients facing preference-sensitive intervention decisions.

In 1982, the President’s Commission for the Study of Ethical Problems in Medicine stated: “Patients who have the capacity to make decisions about their care must be permitted to do so voluntarily and must have all relevant information regarding their condition and alternative treatments, including possible benefits, risks, costs, other consequences, and significant uncertainties around any of this information.”^1^ In 2010, an American Medical Association report states that when there is more than one clinically appropriate course of treatment, the AMA believes shared decision-making, including the use of patient decision aids, has the potential to enhance the overall value of health care in the United States.^2^ In the 30 years in between, many have articulated similar standards for how medical decisions should be made.^3^–^5^

A recent development in thinking about the quality of care is the patient-centered medical home (PCMH). The American College of Physicians and the American Academy of Family Practice have joined their pediatric and osteopathic counterparts to endorse PCMH as a standard of care. Among its tenets are that patients should have a personal physician who ensures that “care is coordinated and/or integrated across all elements of the complex health care system” and that their patients have the opportunity to participate in shared decision making when faced with medical choices. 6

Both prostate surgery for cancer and the elective insertion of stents to treat coronary artery disease are prototypical preference-sensitive interventions. External beam radiation, brachytherapy and conservative management without immediate treatment are all options for most prostate cancer patients. Surgery may offer a slight survival advantage over the alternatives but the serious side effects clearly make this a decision in which patients should play an important role.^7^–^10^ Two recently published analyses indicate there is no survival value of surgery for men over 65^11^ or for men with low-risk cancers.^12^ Percutaneous coronary interventions (PCIs), most commonly insertion of stents, have been shown to provide a survival benefit if they are done within 24 hours of a myocardial infarction (MI).^13^ However, most other patients with heart disease do not derive survival benefit over medical management alone.^14^–^17^ Stenting can provide angina relief, but similar benefits can be achieved with medical management.^17^,^18^ Thus, both interventions, while quite different clinically, are ideal for studying decision making because they have small, if any, mortality benefits over conservative management, potentially significant quality of life implications and no clinical urgency: just the kind of decisions for which informing and involving patients is most important and for which medical homes should be helpful.

A recent survey of Medicare beneficiaries who had undergone either surgery for prostate cancer or elective coronary artery stenting provides an opportunity to examine the current state of informed, ethical decision making for these two procedures.

## METHODS

### Overview

A mail survey was conducted of Medicare beneficiaries whose claims indicated they had either surgery for prostate cancer of elective coronary artery stenting in the last half of 2008. The study was approved by the Institutional Review Boards of the Dartmouth Medical School and the University of Massachusetts Boston and performed in cooperation with the Centers for Medicare and Medicaid Services (CMS).

### Study Samples

Participants had to be enrolled in Medicare Parts A and B with no managed care participation for the previous 12 months and had to be at least 66 years old at the time of the procedure to allow at least one year to check prior claims. Nursing home residents were excluded. Claims-based algorithms were applied to identify potential subjects for the period August 1, 2008 through December 31, 2008. A random sample of 800 patients who had undergone each procedure was selected and sent to CMS, which removed deceased individuals and returned contact information for the rest.

Prostate surgery patients were eligible if they had an inpatient claim for the procedure and evidence of a prostate cancer diagnosis during the admission when the prostatectomy was performed. Stent patients were eligible if they had a Part B physician's claim for a stent procedure or had a hospitalization during which there was an ICD-9 procedure code for a percutaneous coronary intervention (PCI). They were excluded if they were admitted through the emergency department, had either an acute myocardial infarction ( AMI) or an unstable angina code associated with the sampled claim, or had a claim for a PCI or coronary artery bypass during the year preceding the sampled procedure.

### Questionnaire Development

The questions drew heavily on three previous surveys: two surveys of prostate cancer patients^9^,^10^ and the 2008 DECISIONS survey about how nine decisions were made.^19^,^20^ Cognitive testing was done to make sure most respondents understood the questions and that their answers accurately reflected what they had to say.^21^

Four survey questions are the focus of this analysis:

1. Before this (INTERVENTION), did a doctor talk with you about (EACH ALTERNATIVE)?
  - IF YES, Did the doctor talk about (EACH ALTERNATIVE) as a choice to seriously consider?
    Alternatives to prostate surgery were external beam radiation, radioactive seed implants, and not having active treatment right away. The alternatives to stents were CABG and using medicine only.
2. Before the (INTERVENTION) how much did a doctor talk with you about the reasons to have (INTERVENTION)— a lot, some, a little, or not at all?
3. Before the (INTERVENTION), how much did a doctor talk with you about why you might not want to have (INTERVENTION)—a lot, some, a little, not at all?
4. Before this (INTERVENTION) did a doctor ask you if you wanted to have (INTERVENTION) instead of doing something else to manage your (CONDITION)?

### Data Collection Protocols

Selected subjects were first sent a letter signed by the CMS Privacy Officer describing the study and providing a toll-free number to call to opt out. Two weeks later, subjects who had not called were sent a cover letter, a questionnaire, a postage paid return envelope, and a $5.00 cash incentive. This was followed by a postcard reminder, another mailing of the complete packet, and a reminder telephone call for those for whom we could find a number. All respondent contact materials were offered in both English and Spanish.

### Analysis

The coded answers were entered into a data file, then 100% key verified.

To focus on decision making for people likely to have a real choice, in our sampling we attempted to exclude those who had an MI proximate to the stent procedure or who had been admitted through an Emergency Room (ER). There were 23 survey respondents who said they had had a heart attack within a week of the procedure and an additional 98 patients reported that they had been admitted through the ER. These 121 patients were excluded so those analyzed matched those whom studies show on average derive no survival benefit from stents.

The answers to the four questions listed above were used to create a Decision Process Score, giving one point each if the respondent said providers 1) presented at least one alternative to the intervention they received as something to seriously consider, talked about the reasons 2) “for” and 3) reasons “might not want” the intervention “a lot” or “some” and 4) asked what the respondent wanted, yielding a score ranging from zero to four.

There were 21 prostate cancer and 25 stent patients who had three or four values missing from their Decisions Scores, and they are omitted from the analysis. There were 79 (48 prostate cancer and 31 stent) respondents who had one missing value and five who had two missing values. For those missing values, we imputed the mean based on those in the sample who answered the other questions in the Decision Score the way they did.

Three items adapted from the Rose angina questionnaire asked whether or not during the month before the stent procedure respondents had any pain or discomfort in their chest or arm when they: a) did strenuous exercise; b) walked up stairs; c) walked one or two blocks on level ground.^22^ This scale generated a symptom score of 0 to 3. Those who reported pain on at least one item were given 0.5 for each other question they did not answer. We imputed the overall mean for four respondents who did not answer any questions.

The primary analysis consists of descriptive statistics, presenting patient reports related to the decision making process. Two regression models were run predicting the Decision Process Score for each procedure separately that used the demographic characteristics we measured and the measured clinical covariates. STATA version 11 (StataCorp, LP, College Station, TX) was used for the analysis.

## RESULTS

Of the 800 sampled for prostate surgery, three were found to be ineligible because they were deceased or lived in a nursing home. Of the 797 believed to be eligible, 685 returned a survey, a response rate of 86%.

Of the 800 stent patients sampled, 22 were ineligible because they had died, were in nursing homes, or because they reported they had not had a stent procedure near the date indicated in the claims. Of the 778 who were thought to be eligible, 593 returned a questionnaire, a response rate of 76%. Only 472 of those were included in this analysis for reasons outlined above.

We compared respondents and nonrespondents with respect to age, gender and race. There were no differences for either sample with respect to age. Male stent patients responded at a higher rate than females (p = 0.02); white prostate surgery patients responded at a higher rate than nonwhites (<0.001).

The average time between the procedure and receipt of a returned questionnaire was 14 months; that time did not differ by procedure.

Table 1 displays the basic demographic and clinical characteristics of the two samples. The prostate surgery patients were considerably younger than the stent patients, better educated, more likely to be married, and, of course, they were all male, whereas 38% of the stent patients were female. The self-rated health of the prostate surgery patients was better as well.

**Table 1:** Demographic and Clinical Characteristics of Samples by Decision

	PCa Surgery (n=685)†	Stent (n=472)†
	No. (%)	No. (%)
Age		
65-70	342 (52)	114 (25)
71-75	258 (39)	135 (29)
> 76	63 (9)	212 (46)
Gender (Male)	685 (100)	294 (62)
Race (White)	588 (90)	419 (90)
Married/Partner	598 (88)	303 (65)
Education		
HS Grad or less	217 (33)	240 (52)
Some college	181 (27)	111 (24)
College grad +	262 (40)	108 (24)
Self-rated Health		
Excellent	166 (25)	23 (5)
Very Good	297 (45)	120 (26)
Good	158 (24)	197 (42)
Fair or Poor	43 (6)	124 (27)
Cancer Spread Outside Prostate	51 (8)	
Angina Symptom Score		
Zero		235 (54)
1 – 2		94 (22)
3		107 (24)
Had Previous Bypass Surgery		123 (28)
Had Previous Heart Attack		96 (21)
Total Comorbidities‡		
Zero		227 (49)
1		163 (35)
2		55 (12)
3-4		20 (4)

† *The number reported can vary due to item non-response*

‡ *Comorbidities include diabetes, congestive heart failure, COPD, and stroke*

Fifty-four percent of the stent patients reported that they had had no arm or chest pain in the month preceding the sampled stent procedure; 28 percent had had a CABG in the past; 21 percent said they had had a heart attack at some time in the past, although not within a week of the stent procedure. Table 1 also presents the number of four co-morbid conditions (diabetes, heart failure, stroke history or COPD) reported by stent patients; these data were not collected for prostate cancer patients.

Table 2 presents data on how patients reported they interacted with physicians when making the decision to have the interventions. Prostate cancer surgery patients frequently (64%) reported that they were presented with at least one alternative to surgery (brachytherapy, external beam radiation or conservative management) to consider seriously; a third of them said that no immediate active treatment was presented as a serious option. Virtually all (95%) reported discussing the reasons for surgery “a lot” or “some”, while fewer (63%), but still a clear majority, reported “a lot” or “some” discussion of reasons they might not want surgery. Seventy-six percent said their physician asked them what they wanted to do. Prostate cancer surgery patients also were very likely (58%) to have gone on the Internet for information and to report talking with more than one doctor (67%) about the decision, although primary care physicians were seldom (1%) cited as having major input.

**Table 2:** Discussion of Pros and Cons, Sources of Information, Alternatives Considered, and Patient Input

	PCa Surgery (n = 685)†	Stent (n = 472)†
	n (%)	n (%)
Exposure to information about decision		
Doctors discussed “some” or “a lot” :		
Reasons for surgery	625 (95)	341 (77)
Reasons might not want surgery	416 (63)	85 (19)
Patient went on Internet for information	394 (58)	61 (14)
Patient talked with 2 + doctors about decision	443 (67)	128 (29)
Patient talked most with specialist	550 (83)	399 (86)
Patient talked most with primary care physician	9 (1)	14 (3)
Alternative options considered		
Doctor discussed watchful waiting (PCa) or medication management (Stent) as a serious option	221 (34)	25 (6)
Doctor discussed an alternative§ intervention as a serious option	368 (57)	22 (5)
Doctor discussed any alternative§§ as a serious option	408 (64)	43 (10)
Patient’s views reflected in decision		
Doctors asked about patient’s preference for treatment type	497 (76)	69 (16)

† The number reported can vary due to item non-response

§ Alternatives included radiation or brachytherapy for PCa Surgery patients and CABG surgery for stent patients

§§ Alternatives included radiation, brachytherapy, or watchful waiting for PCa surgery patients and CABG surgery or medication management for stent patients

The dynamics of decision making reported by stent patients were quite different. Very few (10%) said they were presented with any alternative approach (CABG or conservative management) as a serious alternative to having a stent; only six percent were offered conservative management as a serious option. While most patients (77%) reported discussing the reasons for the intervention “a lot” or “some” with their physicians, only 19% said their physicians discussed the reasons they might not want the procedure “a lot” or “some”. Only 16% said that they were asked about their own treatment preference. The picture of a minimal decision making process was augmented by the facts that only 14% of stent patients reported going on the Internet for information and only 29% reported talking with more than one physician about the decision. Like prostate cancer surgery patients, stent patients seldom (3%) said a primary care doctor had major input about the decision.

Linear regression models were run to predict the Decision Process Scores for each procedure. The left-hand model in Table 3 for prostate cancer surgery patients shows that there is a significant tendency for patients with more education to report a more substantive decision making process for prostatectomy (p < 0.001); whites (p = 0.04) and those with partners (p = 0.02) also report a more extensive decision making process. However, patient age, self-reported health status, and evidence of cancer spread were unrelated to the Decision Process Score, as was time between surgery and completing the survey

For coronary artery disease, there may be a stronger case for inserting a stent, and hence perhaps less shared decision making, if the patient has severe angina or has had a previous MI or CABG. The stent model in Table 3 shows that patients who had a higher angina symptom score, reported a more (not less) extensive shared decision making process (p = 0.002). No patient demographic characteristics were significantly related to the decision-making process, nor were any of the other measures of their health status or histories, although those with a previous heart attack also tended to report more discussion (p = 0.07).

**Table 3:** Regression Analysis of Decision Process Score

	PCa Surgery Model (n = 664)		Stent Model (n = 447)	
	*β* [95% CI]	p	*β* [95% CI]	p
Intercept	2.13 [1.76,2.50]	<0.001	0.91 [0.53,1.29]	<0.001
Covariates				
Age	0.06 [−0.07,0.18]	0.38	0.01 [−0.10,0.13]	0.83
Gender(Male)			0.07 [−0.12,0.27]	0.48
Race (White vs. Nonwhite)	0.28 [0.01,0.54]	0.04	0.02 [−0.27,0.31]	0.89
Education	0.21 [0.11,0.31]	<0.001	0.04 [−0.07,0.15]	0.45
Married/Partner	0.29 [0.05,0.54]	0.02	0.10 [−0.10,0.30]	0.32
Self-rated Health	0.04 [−0.06,0.14]	0.45	−0.08 [−0.19,0.04]	0.18
Time from Procedure to Survey (Months)	−0.01 [−0.06,0.04]	0.59	0.05 [−0.01,0.10]	0.11
PCa Surgery Covariates				
Cancer Spread	−0.05 [−0.35,0.25]	0.74		
Stent Covariates				
Angina Symptom Score			0.11 [0.04,0.18]	0.002
Had Previous Bypass			0.03 [−0.17,0.24]	0.76
Had Previous Heart Attack			0.20 [−0.02,0.41]	0.07
Total Comorbidities			0.07 [−0.04,0.19]	0.20

## DISCUSSION

The recently published results from the DECISIONS survey reported that patients were largely uninformed and uninvolved in common decisions about long-term medication and cancer screening, though they were somewhat more involved in decisions about back surgery and hip and knee replacement.^20^ When patients have options that deliver similar benefits and entail different risks, as those in this study did, they should have a thorough discussion of their options before treatment decisions are made.

Prostate cancer surgery patients report considerable interaction with physicians around the surgical decision. However, given the limited survival benefit provided by surgery, we think that almost all surgical patients (not just 34%) should be offered conservative management as a serious option, particularly those over 70 and those in poor health. There also was no evidence that those patients over 70 had a more thorough decision making process than others.

Two recent papers report that elective stent patients usually have the misperception that the procedure is extending their lives, even when their physicians do not share that perception.^23^,^24^ This study provides the explanation: there is very little information sharing or discussion with patients when stent procedures are done. It also extends those findings from specific clinical settings to the national Medicare population.

One possible reason for limited discussion is that many stent procedures are done in conjunction with a diagnostic angiogram, limiting the opportunities for discussion. A possible reason those with angina symptoms report a better decision process is that they may have been interacting with physicians about possible options over a longer period of time. However, given the frequency with which stents are placed when angiograms are performed, we would argue that a discussion of the potential decision to insert a stent should be a routine part of the decision to perform a diagnostic angiogram. Another explanation comes from recent focus groups with cardiologists.^25^ While the cardiologists knew that existing clinical studies had found no survival benefit for those with stable angina, many were still convinced of the benefits of PCI, even for asymptomatic patients.

Efforts to improve decision making should continue to focus on increasing patient involvement, but we also think increasing the involvement of primary care providers when decisions are being made would be beneficial. Fewer than three percent of respondents said their primary care provider played a major role in their decisions. Because primary care providers are likely to be less predisposed to a specific treatment than specialists, they may provide more balanced information and may increase the likelihood that conservative options are considered. In addition, there is evidence that patients believe that important decisions are best carried out with their primary care providers.^26^ As patient-centered medical homes become robust centers for care coordination it should become more common for patients to rely on that setting for information and guidance when facing decisions.^27^ The results also point to the potential value of decision aids: routinely providing patients with unbiased, balanced material in print, DVD or web-based form would be a major step to ensure that patients know all the reasonable options and the pros and cons of each.^2^,^28^

The study’s strengths are that data were collected from a national probability sample of Medicare patients who underwent these procedures and the response rates were high. The major limitation is that the data come from patient reports, which may imperfectly reflect the actual interactions with physicians. Also, we surveyed only those who had the procedures. It is possible that those who chose other paths had a better decision making process. However, that does not mitigate the ethical responsibility of providers to inform and involve patients. These patients did get the interventions, and many of them, including the majority of stent patients, were not adequately informed or involved in the decision.

Almost all patients considering prostate cancer surgery should seriously consider radiotherapy and conservative management. Although CABG is often not a reasonable alternative for stent patients like those in this study, all stent candidates with stable angina should seriously consider conservative medical management. The implementation of the PCMH should bring more involvement of primary care physicians in decisions about interventions and increase the likelihood that patients facing major decisions will understand all their options and have their voices heard. The routine use of decision aids would also increase patients’ information about options, though not necessarily their involvement in decisions. Meanwhile, these data provide a challenge and a baseline against which to measure the progress that is very much needed.
